# The MomConnect Nurses and Midwives Support Platform (NurseConnect): A Qualitative Process Evaluation

**DOI:** 10.2196/11644

**Published:** 2019-02-13

**Authors:** Alex Emilio Fischer, Jane Sebidi, Peter Barron, Samanta Tresha Lalla-Edward

**Affiliations:** 1 Wits Reproductive Health and HIV Institute University of Witwatersrand Johannesburg South Africa; 2 HIV/AIDS, TB and MCWH National Department of Health Government of South Africa Pretoria South Africa; 3 School of Public Health University of Witwatersrand Johannesburg South Africa

**Keywords:** evaluation, mHealth, mobile phone, MomConnect, NurseConnect, South Africa

## Abstract

**Background:**

Over the past decade, mobile health has steadily increased in low-income and middle-income countries. However, few platforms have been able to sustainably scale up like the MomConnect program in South Africa. NurseConnect was created as a capacity building component of MomConnect, aimed at supporting nurses and midwives in maternal and child health. The National Department of Health has committed to expanding NurseConnect to all nurses across the country, and an evaluation of the current user experience was conducted to inform a successful scale up.

**Objective:**

This study aims to evaluate the perception and use of NurseConnect by nurses and midwives to produce feedback that can be used to optimize the user experience as the platform continues to scale up.

**Methods:**

We conducted focus group discussions and in-depth interviews with 110 nurses and midwives from 18 randomly selected health care facilities across South Africa. Questions focused on mobile phone use, access to medical information and their experience with NurseConnect registration, as well as the content and different platforms.

**Results:**

All participants had mobile phones and communication through calls and messaging was the main use in both personal and work settings. Of 110 participants, 108 (98.2%) had data-enabled phones, and the internet, Google, and apps (South African National Department of Health Guidelines, iTriage, Drugs.com) were commonly used, especially to find information in the work setting. Of 110 participants, 62 (56.4%) were registered NurseConnect users and liked the message content, especially listeriosis and motivational messages, which created behavioral change in some instances. The mobisite and helpdesk, however, were underutilized because of a lack of information surrounding these platforms. Some participants did not trust medical information from websites and had more confidence in apps, while others associated a “helpdesk” with a call-in service, not a messaging one. Many of the unregistered participants had not heard of NurseConnect, and some cited data and time constraints as barriers to both registration and uptake.

**Conclusions:**

Mobile and smartphone penetration was very high, and participants often used their phone to find medical information. The NurseConnect messages were well-liked by all registered participants; however, the mobisite and helpdesk were underutilized owing to a lack of information and training around these platforms. Enhanced marketing and training initiatives that optimize existing social networks, as well as the provision of data and Wi-Fi, should be explored to ensure that registration improves, and that users are active across all platforms.

## Introduction

As a middle-income country, South Africa has committed to achieving the maternal and child health targets set out in both the Millennium Development Goals and Sustainable Development Goals (SDGs) [1,2]. Over the past decade, great strides have been made, which include the introduction of MomConnect by the South African National Department of Health (NDOH) in 2014 [3,4]. MomConnect is a mobile health (mHealth) solution created to improve and promote maternal health services in South Africa by providing pregnant mothers with free stage-based messaging and a helpdesk, while also creating a universal pregnancy registry [5,6].

Strong government leadership and high mobile penetration [7] have led to over 2 million registered MomConnect users; this success prompted the development of NurseConnect, a complementary platform for health care workers in the maternal and child health space [6]. Like MomConnect, NurseConnect provides its users with short message service (SMS) text messages and access to a helpdesk. In addition, users have access to a mobile website (mobisite) with comprehensive information on themes introduced by the SMS text messages [5,8]. Since its launch in 2016 [9], NurseConnect has >23,000 registered users (Personal communication Jane Sebidi; based on extract from District Health Information System, July 5, 2018), leading the NDOH to consider expanding the platform to engage nurses from all departments, not just maternal and child health [5].

Globally, the World Health Organization (WHO) has identified the health workforce as one of its 6 building blocks for health system strengthening [10], while South Africa’s National Development Plan 2030 highlighted primary health care, with a focus on training and mentoring nurses to build capacity and increase job satisfaction [11]. In 2015, the NDOH incorporated the need for health system strengthening into the South African mHealth Strategy 2015-2019 and recommended that mHealth should be utilized to strengthen health human resources [12], which is what NurseConnect has been developed to do. Many studies have shown the positive effects of mHealth interventions on health care workers in low- and middle-income countries [13-15]; however, many of these platforms were pilot projects that failed to scale up successfully [16,17]. With the NDOH committed to expanding NurseConnect to all nurses across the country [5], and the initial high enrollment numbers [18], NurseConnect has the potential to be successfully scaled up, but the current platform and user experience must be evaluated to identify possible challenges going forward [13,18]. Therefore, a mixed-method process evaluation was undertaken. In this paper, we present the qualitative aspects only aimed at evaluating the perceptions and use of NurseConnect by health care workers to provide feedback to optimize the user experience to inform the expansion of NurseConnect.

## Methods

The NurseConnect evaluation in this paper specifically refers to the collection and evaluation of qualitative data from focus group discussion (FGDs) and in-depth interviews (IDIs) with nurses and midwives from selected facilities across South Africa. Of note, this paper does not evaluate any quantitative data pertaining to the outcomes or effectiveness of the NurseConnect platform, as these finding will be the focus of a separate publication.

### NurseConnect Content

The NurseConnect platform was based on the Integrated Behavioral Model and Adult Learning Theory of change, where evidence indicates that engagement is the key to absorbing information from Web-based learning situations. Health care worker training and mentoring specialists compiled and designed the content with input from local doctors and nurses in the maternal health field. SMS text messages were presented in concise, simple language 2-3 times a week and often contained links to expanded papers on the mobisite. The mobisite could also be reached directly from the internet, and the helpdesk could be activated by responding to any of the SMS text messages. More recently, where nurses have smartphones, SMS text messages have been replaced by WhatsApp messages.

**Figure 1:**
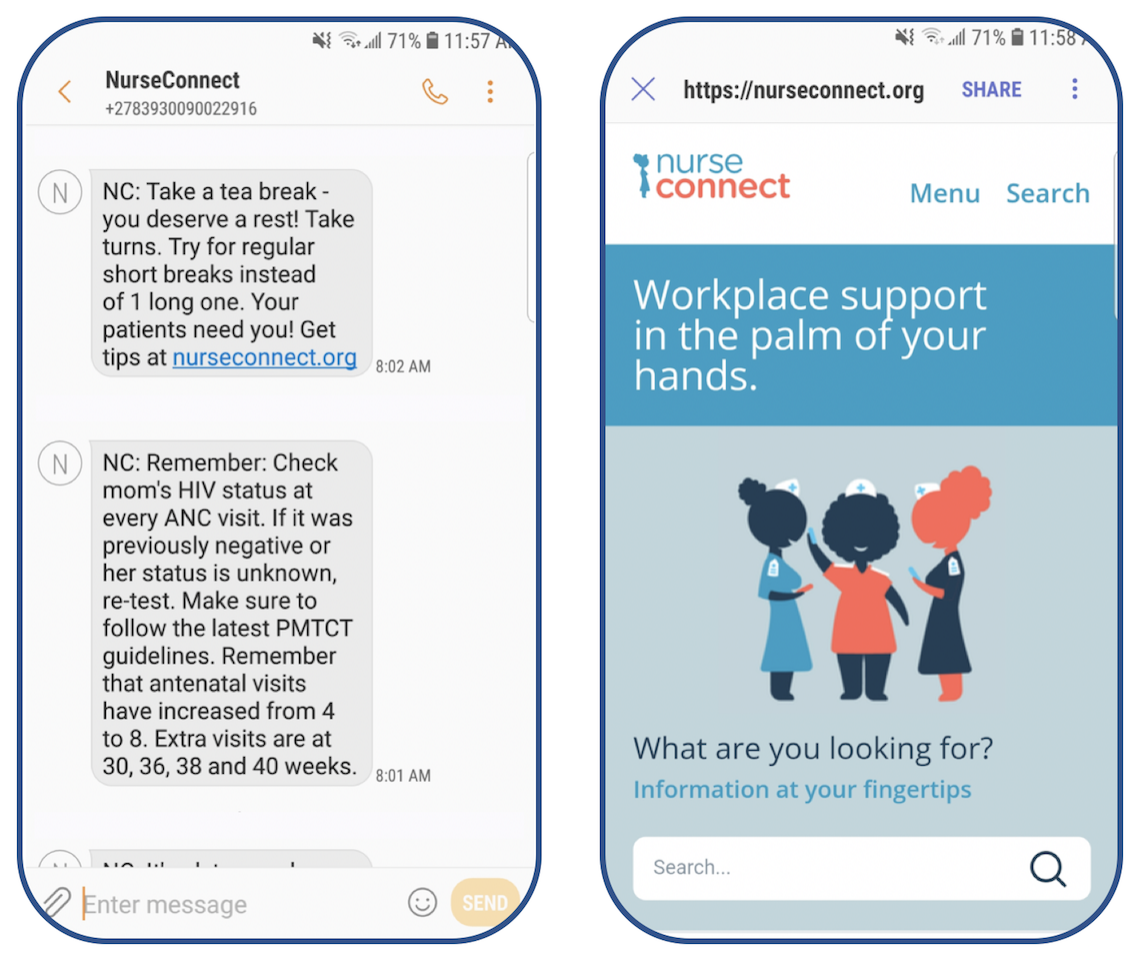
NurseConnect sample messages and mobisite.

The content was divided into 2 main categories—informational and motivational. The informational content aimed to improve users’ knowledge of maternal and child health, while the motivational content aimed to inspire users to make small actionable changes to increase productivity and happiness in their work. Figure 1 displays example messages, as well as a screenshot of the NurseConnect landing page.

### Setting

**Figure 2:**
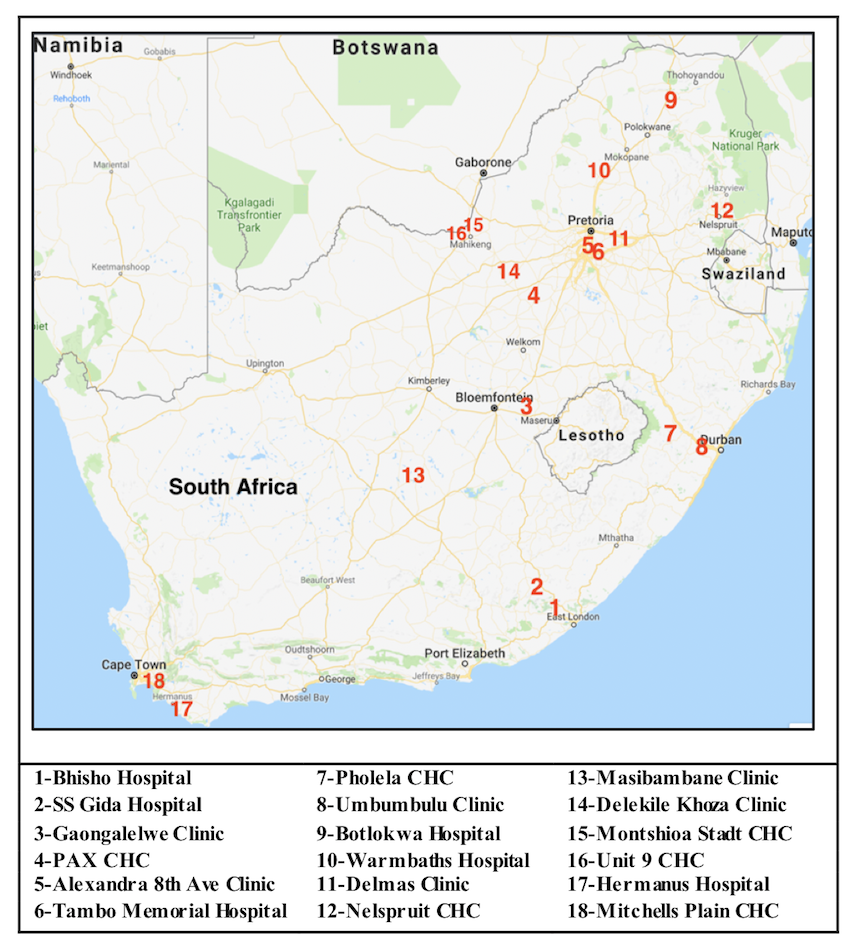
Facility locations. Ave: avenue; CHC: community health center.

To minimize bias, we randomly chose 18 facilities to equally represent a national population by ensuring that all provinces, types of facilities (ie, hospitals, clinics, and community health centers [CHCs]), and regions (ie, urban, periurban, and rural) were included. Figure 2 details the names and locations of these 18 facilities.

After provincial and district approval were obtained, facility visits were scheduled. Each facility was asked to provide a group of 6-8 registered staff to participate in an FGD or IDI, as well as a private room for the discussions to take place. Each site visit lasted from half a day to 3 days. Each FGD and IDI took between 15 minutes and 1 hour, depending on the operational demands of the facility and the active participation of the staff.

### Data Collection

All 18 approached facilities consented to participate in the research, and the site visits were conducted between December 12, 2017 and April 10, 2018. Convenience sampling provided a total of 110 nurses and midwives, who participated in the focus groups and interviews.

Using a pretested and piloted guide, 2 experienced moderators facilitated the FGDs and IDIs, with the principal investigator being present for 15 of the 18 site visits. The decision to conduct an FGD or IDI depended on the number of available NurseConnect registered participants present at the facility during the data collection visit. The FGDs and IDIs were audiorecorded so that the conversations could later be transcribed, and the facilitator also documented notes after discussions. Although an option of answering in vernacular was presented, participants elected to speak English.

### Data Analysis

A total of 18 FGDs and 9 IDIs were conducted during the site visits. All these recordings were transcribed by the principal investigator into Microsoft Word documents. A selection of transcripts was randomly selected and independently verified by another team member to ensure accuracy. A code list agreed upon by the evaluation team was constructed to define relevant codes and emergent themes. The transcripts were then uploaded to MAXQDA V 1.2 (Verbi Software) and investigated with the code list. All transcripts were coded by the principal investigator, while a selection of transcripts was independently coded by another study team member to ensure consistency. Upon completion, each code was concentrated into data reduction tables, then further refined into summary tables for reporting. Data were summarized into the themes of mobile use, registration, platforms, and user experience and content.

### Ethical Consideration and Approval

Ethics approval for this evaluation was obtained from the University of the Witwatersrand Human Research Ethics Committee (M106976) on October 21, 2016. Participation in the data collection was voluntary. Consent forms were signed for both participation and voice recording.

## Results

### Mobile Phone Use

All 110 participants stated that they had personal mobile phones; 4 also had access to a mobile phone provided by work. Two participants had basic phones, some had feature phones (access to data and some apps like the internet and WhatsApp), and the majority used smartphones.

**Figure 3:**
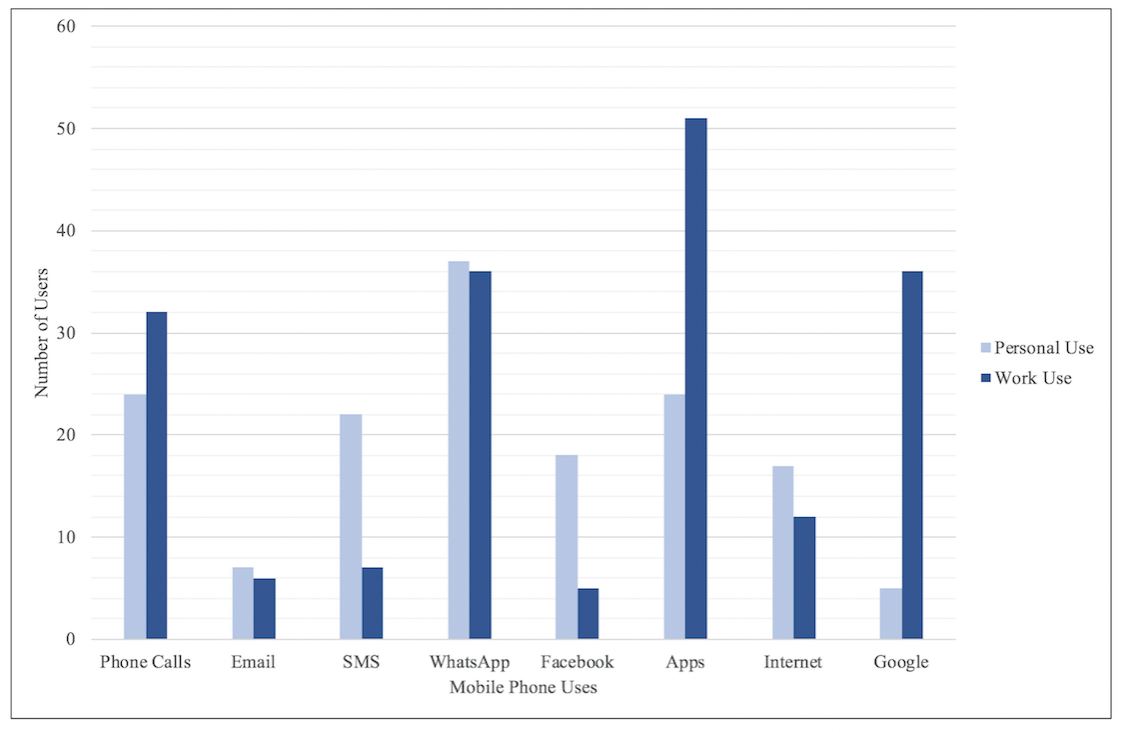
Common mobile phone uses. SMS: short message service.

The most common personal use of phones was for communication, with the majority of participants using WhatsApp (often in group chats), SMS text messages, and phone calls to stay in touch with their friends and family, as well as their work and their children’s schools. In addition, Facebook was a popular form of communication and networking, as participants could interact with nursing communities on a national or global scale such as the International Midwives Page. Furthermore, participants used the internet and Google, as well as a variety of apps, which included the camera, banking, GPS or navigation, voice or video recordings, educational apps, gaming, and YouTube (Figure 3).

Some participants stated that high data costs prevent them from using their phones for work, but most participants were active phone users and took advantage of this resource to help them with their health care in a variety of ways (Figure 3). Communication was the main use, with many participants calling or messaging their colleagues when they needed assistance. Most interactions on WhatsApp were made in groups though, with many participants belonging to a number of health-related groups; these groups consisted of colleagues, as well as a variety of other health care professionals, and these different clusters allowed participants to share media, ask questions, and support each other.

So, we use WhatsApp. We just shoot the picture, neh, of the X-ray, then we just send it to that doctor, maybe the specialist, and then that specialist will respond.

Nurse, rural hospital

In addition, participants frequently used their phones to look up clinical information about symptoms, diagnoses, and medications, stating that they were generally able to find the information that they required. The main source of information was the internet through Google; however, some participants were weary of the information found over the Web and preferred to use trusted medical apps, like the NDOH guidelines, iTriage, Medscape, Drugs.com, and Drugs Dictionary.

Websites, you know, the internet is full of…it’s not authentic, because they are not journals per se. So, you need things that are authentic because we are dealing with people, you know what I mean, not robots here. We don’t do trial and error here. We need to be 100% correct.

Nurse, urban clinic

### NurseConnect

#### Registration

Of 110 participants, 62 (56.4%) were registered to NurseConnect, and many of them had been registered for over a year (Table 1). The absence of more registered participants was mainly attributed to a lack of awareness of the NurseConnect platform, which included a number of health care workers who thought that NurseConnect and MomConnect were the same product.

With me, this is the first time. I even though you wanted to write about MomConnect, when the sisters said it was NurseConnect, so this was the first time.

Nurse, rural CHC

**Table 1:** Facilities sampled and the number of participants per facility.

Facility	Province	Total participants	NurseConnect registered participants, n (%)
Bhisho Hospital	Eastern Cape	2	2 (100.0)
SS Gida Hospital	Eastern Cape	15	6 (40.0)
Gaongalelwe Clinic	Free State	8	6 (75.0)
PAX Community Health Center	Free State	1	1 (100.0)
Alexandra 8th Avenue Clinic	Gauteng	1	1 (100.0)
Tambo Memorial Hospital	Gauteng	7	0 (0.0)
Pholela Community Health Center	KwaZulu-Natal	7	5 (71.4)
Umbumbulu Clinic	KwaZulu-Natal	13	12 (92.3)
Botlokwa Hospital	Limpopo	6	6 (100.0)
Warmbaths Hospital	Limpopo	7	7 (100.0)
Delmas Clinic	Mpumalanga	3	3 (100.0)
Nelspruit Community Health Center	Mpumalanga	2	2 (100.0)
Masibambane Clinic	Northern Cape	1	1 (100.0)
Delekile Khoza Clinic	North West	7	2 (28.5)
Montshioa Stadt Community Health Center	North West	6	0 (0.0)
Unit 9 Community Health Center	North West	8	2 (25.0)
Hermanus Hospital	Western Cape	13	3 (23.0)
Mitchells Plain Community Health Center	Western Cape	3	3 (100.0)
Total	Not applicable	110	62 (56.4)

The NurseConnect registration process was described as quick and easy by the majority of participants, with only a few of them stating that they had complications because of phone issues or difficulties with the clinic code. When asked about the initial sign-up process, there seemed to be 2 main ways that participants became aware of NurseConnect and were signed up. The first was during visits from an external representative familiar with NurseConnect (ie, District Clinical Specialist Team Midwife, NDOH representative, Ideal Clinic Representative) who shared the registration process with staff during their visits. The second method of NurseConnect registration was when facilities had an internal NurseConnect representative that went to a NurseConnect training workshop or in-service, then brought the information back to connect their fellow staff members.

In addition, unregistered participants were probed about why they had never signed up, and most of them stated that they had never heard of NurseConnect before, or thought that it was referring to MomConnect. For those familiar with the platform, some of them were not working the day that their facility was registered, while others cited data and time constraints as the main barriers. Some participants were not allowed to have phones at work, while others mentioned message fatigue, lack of personal motivation, or being too junior as reasons not to register.

Like for instance, we are not being given, offered data. Like when you read message, it consumes data, and we are getting this every day. I would appeal to the Department to give us the data to those that are NurseConnected.

Nurse, rural clinic

#### Platforms and User Experience

All registered participants stated that they received the NurseConnect messaging, and all but one stated that they received as SMS text message. Participants liked receiving the SMS text messages because they were free and easy to access, even for people who were not very technology-savvy or did not have adequate data. Some participants saved the SMS text messages so that they could refer to them at a later date, while others forwarded the SMS text messages to friends and colleagues who were not registered.

I will say SMS, you just read it, you don’t need data, you don’t need anything. Your phone needs to be just charged, that’s all.

Nurse, urban hospital

I sent it to my sister, I think it is the one she is talking about. The one with after work, the legs, my sister is a nurse as well, and working in maternity. So, I forwarded it to her.

Nurse, periurban hospital

Only 1 participant was receiving messages through WhatsApp, and none of the other participants knew that this was an option. When asked about the possibility of accessing NurseConnect through WhatsApp, participants were evenly divided, with some against the idea, while others were open to it. Participants who were open to using WhatsApp for NurseConnect stated that they liked the user-friendly interface and suggested that WhatsApp groups could be leveraged to create discussions and ask questions about topics, instead of the one-way flow of information from SMS text messages.

So, if we can then get connected on it, and then maybe open up a platform like a WhatsApp where people can then also interact about those messages and then take discussions further.

Nurse, urban clinic

Participants who did not want to use WhatsApp for NurseConnect stated that data costs were the main barrier, while others viewed WhatsApp as a personal platform and did not want to use it for work.

My WhatsApp is more like my personal, personal people only, like close family and friends. I prefer SMS.

Nurse, urban hospital

Of 62 registered NurseConnect users, only 2 stated that they had logged on to the NurseConnect mobisite. One participant stated that she enjoyed the mobisite and searched it for information when they were bored. Another participant stated that she followed the blue link from the SMS text message to get more information; however, she was not familiar with the term “mobisite” and had never explored the site. Four other participants stated that they had clicked on the NurseConnect links in the SMS text messages but were also not aware that the linked paper was on a NurseConnect mobisite. Some other participants stated that even if they were familiar with the site, data costs would still prevent them from using it.

We didn’t hear about the mobisite. That means at the time when she went for the in-service, I don’t think that the mobisite was mentioned.

Nurse, rural clinic

That’s the big thing. That’s the issue why we don’t go to those ”dot com.” The data.

Nurse, rural clinic

None of the registered NurseConnect users who participated had ever used the helpdesk, and only a few of them knew that it existed. One person mentioned that she had received an SMS text message explaining that they could respond but had never tried. Another person had heard of the helpdesk but was under the impression that it would be a call center and did not know that it was active or that it could be accessed via SMS text messages.

We don’t know whether to respond back, so we are not responding back. That is why I am saying it is one-sided. It is giving you the information, you don’t have any chance to, to share your problem.

Nurse, rural hospital

The main barrier to the helpdesk and mobisite was that participants did not know they existed; however, once informed, participants liked the idea and said that they would like to try them. Another barrier to use that many participants mentioned was the data costs of accessing a mobisite or sending an SMS text message.

The problem is like sister said, we don’t have data. That’s the problem, it is why we don’t respond to the messages. Because of money.

Nurse, rural hospital

I think that if we had free Wi-Fi here in the hospital, like if you want to access any information online you don’t have to use your data because it’s work related.

Nurse, urban hospital

#### Content

Participants generally liked the language, structure, and frequency of the NurseConnect messages, stating that they were easy to understand and relevant to their daily work. In addition, they preferred English messages, as the medical content is easier to read and understand in English than traditional languages.

And the language is simple and straight forward. There is no bombastic words like when we were at schools. So, the messages are straight up.

Nurse, rural clinic

It is easy for me. Because it is English. And all of us speak English and it is, it’s the terms that we are using in our profession.

Nurse, rural hospital

If it was like 20 messages a week, then I would get a bit upset because I would think it’s too much. And it would just be taking up all the space on my phone, and I don’t think I would read all of it then. Where if it was less messages every day, or every second day or third day, I would actually take the time and read it, and then maybe even forward it to someone.

Nurse, urban hospital

Messages about the listeriosis outbreak and motivation or stress relief were favored by many, and some participants stated that these messages created behavioral changes. The listeriosis information prompted one facility to conduct a full in-service based on the topic, while another facility improved their temperature-taking practices after receiving the messages. Some participants stated that the motivational messages led them to eat and rest properly during long shifts and before nightshifts, while others greatly appreciated the encouraging messages.

I can give an example, the other day, and I was so happy. I was, the whole week I was teaching patients on HIV, and when the message comes and say your patients are happy about the information that you give them, I was like wow, thank you, talking to me. And I was happy!

Nurse, periurban hospital

Some participants suggested that the topics be expanded to include pediatrics, chronic diseases, geriatrics, and mental health; however, they generally found the messages very informative and helpful. Furthermore, participants suggested that the government could share updated policies, guidelines, and nursing standards through the NurseConnect platform.

What I noticed is that I think you mostly on babies and pregnant women, but there’s nothing about paediatrics, chronic diseases, of geriatrics.

Nurse, rural clinic

There is no need for us to be going on apps and downloading things, let the government give us what they want us to have. The latest information, you know.

Nurse, urban hospital

## Discussion

### Principal Findings

This is the first report describing and evaluating the rollout, uptake, and utilization of NurseConnect in South African public health facilities and the findings show that there were no marked variations between nurses from different provinces, facility types, or regions. High smartphone saturation, coupled with the nurses’ use of WhatsApp, the internet, and various medical apps, reinforced the need to provide quality mHealth solutions that are tailored to the local context. The smartphone infiltration and extensive use of WhatsApp for personal and medical communication suggested that WhatsApp should be encouraged as a NurseConnect channel. To distinguish this platform from SMS text messaging, media messages (voice notes, pictures, and video clips) could be offered as a way to enhance the user experience.

Only 56.4% (62/110) of participants were registered on NurseConnect. The nurses who were not registered exposed gaps that should be addressed to successfully scale up the platform toward 2020 [5,9]. An extensive mixed media marketing campaign should be used to create awareness for the platform, while existing social networks on Facebook and WhatsApp should also be leveraged to spread awareness [5]. The need for marketing initiatives and platform training was reinforced by the lack of mobisite and helpdesk use. Many participants stated that they were not aware of these platforms before the site visits were conducted. Participants thought these platforms would be useful; however, the term “mobisite” was unfamiliar with participants, and “helpdesk” was associated with call-in services, not a 2-way messaging service. Some participants did not trust information found over the Web and preferred using reliable apps, so rebranding the mobisite as a mHealth app, could increase user activity.

I think for an example, in Facebook, there are so many groups including those who are nurses, midwives, primary health care. So, maybe one could paste on Facebook to say there is NurseConnect, this is what they say guys, sign in and let’s get, let’s make it viral.

Nurse, urban CHC

Similar to the internal NurseConnect representatives, who registered their colleagues, high-level users can be identified on these networks and trained as ambassadors or mentors to create demand for NurseConnect, facilitate discussions, and assist with registrations [11]; this bottom-up approach should foster peer-to-peer interactions, while also providing users with a contact to facilitate awareness and training for new platforms [16].

Aside from the lack of awareness of the NurseConnect platform, the biggest barrier to NurseConnect registration, uptake, and utilization remains the cost of data. Data cost was mentioned in almost every part of the discussions, as South Africa has the highest data charges out of the 6 largest Telcom markets in Africa. Data charges in South Africa have been the cause of much debate and protests by the majority of citizens not just these health care workers, making this a very relevant practical (possibly longer term) challenge, which needs to be addressed [19]. As the NDOH prepares to scale up the provision of facility mobile phones, subsidized data or airtime or free Wi-Fi in health care facilities will need to be considered.

The expansion of NurseConnect to all nurses also provides an opportunity to develop and tailor new message streams beyond maternal and child health. Participants suggested incorporating information about noncommunicable diseases and mental health, which are in-line with the WHO country strategy for South Africa [20].

### Limitations

This study has some limitations. While we endeavored to collect information from as many NurseConnect registered users as possible, we ended up with a smaller number than anticipated owing to the health care worker confusion around NurseConnect and MomConnect. Although the identification of the confusion was a valuable finding in itself, it did limit the amount of information we could collect about the NurseConnect implementation. Second, we did not collect sociodemographic information, which may have assisted in contextualizing some of the results or opinions presented. Third, although we collected information from different types of health care facilities across the country, the results presented are not conclusively representative of the entire NurseConnect registered health care population and may lack some differing opinions and experiences of users from facilities that did not participate in the evaluation. Finally, this was a qualitative evaluation that independently investigated health care workers’ perceptions and uses of NurseConnect, without examining the efficacy and outcomes of the platform.

### Conclusions

After discussing NurseConnect with nurses from all 9 provinces of South Africa, it is evident that the platform is not just useful but well-liked by nurses and midwives who use it. Participants found the messages informative, and their suggestions on expanding the content showed that they have been engaging with the platform and felt that it would continue to help them learn and grow. While this positive feedback reinforced the fact that this platform works, this evaluation has also identified gaps and areas of improvement that can be turned into strengths as the NDOH continues to rollout NurseConnect to all nurses across the country by 2020.

Across many facilities, it was discovered that the NurseConnect brand needs strengthening. The lack of awareness surrounding the WhatsApp messages, mobisite, and helpdesk presents an excellent opportunity for growth, as many participants liked these ideas but were previously unaware that platforms other than SMS text messaging existed. The other main barriers to these platforms were time constraints and data costs. The promotion of a zero-rated app and messaging, as well as free Wi-Fi for facilities, would help overcome the data barrier. By combating these barriers and leveraging existing mobile social networks to increase registration and awareness, NurseConnect should continue to develop as the platform becomes available to all nurses across the country.

## Abbreviations

CHC: Community Health Center
FGD: focus group discussion
IDI: in-depth interviews
NDOH: National Department of Health
SMS: short message service
WHO: World Health Organization
